# A soluble biocompatible guanidine-containing polyamidoamine as promoter of primary brain cell adhesion and *in vitro* cell culturing

**DOI:** 10.1088/1468-6996/15/4/045007

**Published:** 2014-08-20

**Authors:** Noemi Tonna, Fabio Bianco, Michela Matteoli, Cinzia Cagnoli, Flavia Antonucci, Amedea Manfredi, Nicolò Mauro, Elisabetta Ranucci, Paolo Ferruti

**Affiliations:** 1 NeuroZone, Viale Ortles 22/4, I-20129 Milano, Italy; 2 Department of Medical Biotechnologies and Translational Medicine, Humanitas Clinical and Research Center, Università degli Studi di Milano, Via Manzoni 113, I-20089 Rozzano, Milano, Italy; 3 Fondazione Filarete, Viale Ortles 22/4, I-20139 Milano, Italy; 4 Department of Chemistry, Università degli Studi di Milano, Via C. Golgi 19, I-20133 Milano, Italy; 5 Consorzio Interuniversitario Nazionale per la Scienza e Tecnologia dei Materiali, Via G. Giusti 9, I-50121 Firenze, Italy

**Keywords:** polyamidoamines, guanidine-containing polyamidoamine, primary brain cells, adhesion promoters, cell culturing

## Abstract

This paper reports on a novel application of an amphoteric water-soluble polyamidoamine named AGMA1 bearing 4-butylguanidine pendants. AGMA1 is an amphoteric, prevailingly cationic polyelectrolyte with isoelectric point of about 10. At pH 7.4 it is zwitterionic with an average of 0.55 excess positive charges per unit, notwithstanding it is highly biocompatible. In this work, it was found that AGMA1 surface-adsorbed on cell culturing coverslips exhibits excellent properties as adhesion and proliferation promoter of primary brain cells such as microglia, as well as of hippocampal neurons and astrocytes. Microglia cells cultured on AGMA1-coated coverslips substrate displayed the typical resting, ramified morphology of those cultured on poly-L-lysine and poly-L-ornithine, employed as reference substrates. Mixed cultures of primary astrocytes and neuronal cells grown on AGMA1- and poly-L-lysine coated coverslips were morphologically undistinguishable. On both substrates, neurons differentiated axon and dendrites and eventually established perfectly functional synaptic contacts. Quantitative immunocytochemical staining revealed no difference between AGMA1 and poly-L-lysine. Electrophysiological experiments allowed recording neuron spontaneous activity on AGMA1. In addition, cell cultures on both AGMA1 and PLL displayed comparable excitatory and inhibitory neurotransmission, demonstrating that the synaptic contacts formed were fully functional.

## 1. Introduction

*In vitro* culturing of neural cells has long since been employed for studying brain metabolism, isolated from influence by the whole organism [1]. Brain cells, including astrocytes, microglia, hippocampal- and cortical primary neurons, require adhesion promoters to facilitate cell attachment, spreading, growth and morphological development. Moreover, improving cell adhesiveness to biomaterials is a major challenge in neural tissue engineering and represents a crucial issue for the development of implanted neural prostheses, biosensors and neural networks [2]. Extracellular matrix (ECM) substances such as collagen and laminin promote axonal regeneration, differentiation, adhesion and migration in the central nervous system and may be defined as the neural cell adhesive substances par excellence [3]. Synthetic biomaterials may be modified with short recognition motifs that mimic the ECM capability of promoting cell binding. Among these, the *arg-gly-asp* (RGD) sequence found in collagen, laminin and fibronectin has been identified as the minimum cell recognition sequence mediating adhesion of many cell types, including neurons [4]. In addition, surface-coating with basic synthetic polypeptides, such as for instance poly-L-lysine (PLL) [1], poly-D-lysine (PDL) [5] and poly-L-ornithine (PLO) [6], as well as polyethyleneimine (PEI) [7], has been shown to promote neural adhesion and enhance neurite extension *in vitro* via a non-receptor-mediated cell binding mechanism, consisting of electrostatic interactions with the negatively charged cell membranes. Most synthetic polycations are cytotoxic and, therefore, need to be removed by washing prior to cell seeding and, for the same reason, are unsuitable for *in vivo* applications.

**Figure 1.**
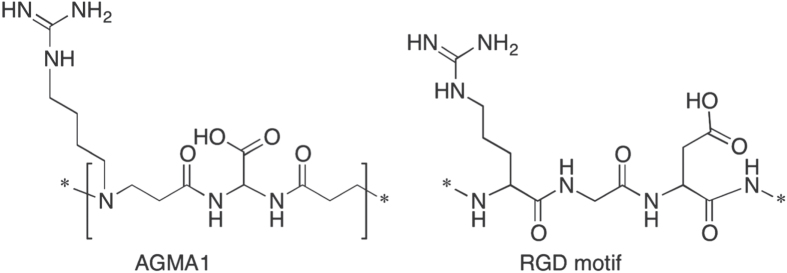
Chemical structure of AGMA1 repeating unit and RGD motif.

Polyamidoamines (PAAs) are linear or crosslinked synthetic *tert*-amine polymers obtained by stepwise polyaddition of *prim-* or *sec*-amines to bisacrylamides [8]. The reaction typically occurs in aqueous solution under mild and environmentally friendly conditions. Many additional functions besides the chain *tert*-amine groups that are a fundamental of PAA structure can be introduced as side substituents. As a rule, PAAs are much less cytotoxic than PLL, PDL and PEI [9, 10]. In previous research, a guanidine-substituted amphoteric, but prevailingly basic PAA named AGMA1, whose repeating unit (figure 1) is reminiscent of the RGD motif, was prepared and studied [11–13]. AGMA1 proved able to enter cells and act as nucleic acid carrier and transfection promoter without exerting any appreciable cell-membranolytic activity even at pH < 5, where its excess positive charges per unit is ≽ 1. In aqueous media, cross-linked AGMA1 formed swollen hydrogels able to promote cellular adhesion and proliferation [14, 15] and were successfully tested as bioresorbable tubular scaffolds for peripheral nerve regeneration in rats [16, 17]. These results were consistent with the hypothesis that AGMA1, owing to its cationic nature and RGD-like structure, was endowed with cell-adhesive properties. Hence, it was deemed not unreasonable to predict that soluble AGMA1, once adsorbed on cell-culture plates, acted as a biocompatible neuronal cell adhesion and proliferation promoter. The aim of this paper is to report on this issue.

## 2. Experimental section

### 2.1. Characterizations

^1^H and ^13^C NMR spectra were run on a Brüker Advance 400 spectrometer operating at 400.132 (^1^H) and 100.623 (^13^C) MHz. Size exclusion chromatography (SEC) traces were obtained with a Knauer Pump 1000 equipped with a Knauer Autosampler 3800, TSKgel G4000 PW and G3000 PW TosoHaas columns connected in series, Light Scattering (LS) Viscotek 270 Dual Detector, UV detector Waters model 486, operating at 230 nm, and a refractive index detector Waters model 2410. The mobile phase was a 0.1 M Tris buffer pH 8.00 (0.05 with 0.2 M sodium chloride. The flow rate was 1 mL min^−1^ and sample concentration 1% w w^−1^. All solvents and reagents, where not otherwise specified, were analytical grade Fluka reagents used as received.

### 2.2. AGMA1 synthesis

AGMA1 was prepared by a previously reported procedure [11]. Briefly, Agmatine sulfate (6.000 g, 25.5 mmol) and lithium hydroxide monohydrate (1.08 g, 25.5 mmol) were added to a solution of 2,2-bisacrylamidoacetic acid (5.07 g, 25.5 mmol) and lithium hydroxide monohydrate (1.08 g, 25.5 mmol) in distilled water (8.5 mL). This mixture was maintained under a nitrogen atmosphere and occasionally stirred for 240 h. After this time, it was diluted with water (8.5 mL), acidified with 1 M hydrochloric acid to pH 4–4.5, and then ultrafiltered through a membrane with nominal cutoff 10000. The fraction retained was freeze-dried. The product was a white powder. Yield: 5.34 g. ^1^H NMR (D_2_O, *δ*): 1.61 (br, NHCH_2_CH_2_CH_2_), 1.76 (br, NHCH_2_CH_2_), 2.79 (br, NHCOCH_2_CH_2_), 3.19 (m, NHCH_2_, NCH_2_), 3.44 (br, NHCOCH_2_), 5.55 (s, CHCOOH). ^13^C NMR (D_2_O, *δ*): 22.3 (NHCH_2_CH_2_CH_2_), 25.0 (NHCH_2_CH_2_), 28.9 (NHCOCH_2_CH_2_), 40.4 (CH_2_NCH_2_), 49.1 (NHCOCH_2_), 52.5 (NHCH_2_), 56.0 (COOHCH), 155.1 (NH_2_CNNH), 171.3 (NHCO), 173.5 (CHCOOH).

### 2.3. Preparation of PLL, PLO and AGMA1 coated plates

Glass coverslips were incubated overnight at 37 °C in 65% nitric acid to make surface rough for cell attachment, then washed in water until neutral pH. Subsequently, they were dry sterilized at 180 °C and placed in a sterile multiwall. Stock solutions of poly-L-lysine (molecular weight 400 KDa, Sigma Chemical), poly-L-ornithine hydrobromide (molecular weight 30–70 KDa, Sigma Chemical) and AGMA1 were prepared in milli-Q sterile water, filtered through a 0.22 *μ*m filter (Millipore) and then diluted to 1 mg mL^−1^. Coverslips were coated by aseptically dispensing 200 *μ*L adhesion promoter solution. After incubating 1 h at ambient temperature in a humidified incubator, the solution was removed, the coverslip rinsed once with sterile water and finally dried overnight at room temperature.

### 2.4. Primary cell cultures

Primary neuronal cultures were prepared from the brains of 18-day-old rat embryos (Charles River, Milan, Italy) as previously described [18], with minor modifications. Briefly, the hippocampi or cortices were isolated from total brain, incubated with trypsin at 37 °C, and then dissociated to obtain separated cells, and grown in neurobasal medium supplemented with B27, 0.5 mM glutamine and 12.5 *μ*M glutamate. Hippocampal neural and astrocytic cultures from embryonic rat pups (E21) were obtained using previously described methods [19]. Briefly, after dissection, the hippocampi/cortices were dissociated by treatment with trypsin (0.25% for 10 min at 37 °C) followed by fragmentation with a fire-polished Pasteur pipette. The dissociated cells were grown in minimum essential medium (Invitrogen, Italy) supplemented with 20% fetal bovine serum (Euroclone, UK) and glucose at a final concentration of 5.5 g L^−1^. After three weeks, in order to collect microglial cells, the astrocyte cultures were shaken on a rotary shaker (200 rpm) at room temperature for 30 min. Microglial cells were maintained as described [20].

All efforts were made to minimize animal suffering and to reduce the number of animals used, in accordance with the European Communities Council Directive of 20 September 2010 (2010/63/UE). All procedures involving animals were performed according to the guidelines of the Institutional Animal Care and Use Committee of the University of Milan.

### 2.5. Immunofluorescence staining of cultured neurons

Neuronal cell cultures were fixed for 20 min at room temperature with 4% paraformaldehyde in in 0.12 M phosphate buffer containing 0.12 M% sucrose. The following antibodies were used: mouse anti-GFAP (Sigma Aldrich, St. Louis, MO) 1:500; anti-tubulin *β*-III, clone TU-20 (Merck Millipore) 1:100; anti-MAP-2 (Merck Millipore), anti-TI-VAMP-2 (produced by T Galli, INSERM, Paris), rabbit anti-SV2a (1:1000; Synaptic System, Goettingen, Germany); mouse anti-MAP2 (Synaptic System GmbH, Goettingen, Germany), 1:300; guinea pig anti-Bassoon (1:300; Synaptic System, Goettingen, Germany), mouse anti-PSD-95 (1:400; UC Davis/NIH NeuroMab Facility, CA, USA). Secondary antibodies were conjugated with Alexa-488, Alexa-555 or Alexa-633 fluorophores (Invitrogen, San Diego, CA, USA). Images were acquired using a Leica SPE confocal microscope. Pixel size was 94.8 nm × 94.8 nm, and acquisition parameters (i.e., laser power, gain and offset) were kept constant among different experimental settings. Images were analyzed with ImageJ software. Colocalization of two or three selected markers was measured using the boolean function ‘and’ for the selected channels. The resulting image was binarized and used as a colocalization mask to be subtracted to single channels. The area of the puncta resulting from colocalization mask subtraction were measured for each marker. A colocalization ratio was set as area of colocalizing puncta/total puncta area number. The total area of the measured synaptic puncta represents synaptic area.

### 2.6. Electrophysiology

Whole-cell patch-clamp recordings were obtained from 14 div neurons with an Axopatch 200B amplifier and pClamp-10 software (Axon Instruments, Foster City, CA). Recordings were performed in the voltage-clamp mode. Currents were sampled at 2 kHz and filtered at 2–5 kHz. External solution [Krebs’-Ringer’s-HEPES (KRH)] had the following composition (in mM): 125 NaCl, 5 KCl, 1.2 MgSO_4_, 1.2 KH2PO_4_, 2 CaCl_2_, 6 glucose, and 25 HEPES-NaOH, pH 7.4. mEPSCs were recorded in the presence of 1 *μ*M tetrodotoxin (TTX). Recording pipettes were fabricated from capillary glass (World Precision Instruments) using a two stage puller (Narishige, Tokyo, Japan) and had tip resistances of 3–5 M when filled with the intracellular solution of the following composition (in mM): 130 K-gluconate (or Cs-Gluconate for IPSCs), 10 KCl, 1 EGTA, 10 HEPES, 2 MgCl_2_, 4 MgATP, and 0.3 Tris-GTP. Neurons were held at −70 or +10 mV to identify, respectively, excitatory or inhibitory miniature events. Recordings were performed at room temperature. Off-line analysis of mEPSCs and mIPSCs have been performed using Clampfit-pClamp-10 software and events had to exceed a threshold of two times the SD of the baseline noise.

## 3. Results and discussion

The present paper relates to the use of a water-soluble peptidomimetic PAA named AGMA1 (figure 1), prepared by polyaddition of 4-aminobutylguanidine (agmatine) to 2,2-bis(acrylamido)acetic acid, as cell adhesion, proliferation and differentiation promoter for *in vitro* culturing of brain primary cells. The repeating unit of AGMA1 is depicted in figure 1. It contains three ionizable groups (carboxyl-, *tert*-amine- and guanidine) with pK_a_ values 2.25, 7.5 and >12.1, respectively [11]. AGMA1 is amphoteric, but prevailingly basic, with isoelectric point of ∼10. At pH 7.4 it bears, on average, 0.55 excess positive charges per unit. Notwithstanding, it is highly biocompatible both *in vitro* (IC_50_ ≽ 5 mg mL^−1^ towards several cell strains) and *in vivo* (MTD in mice >0.5 g Kg^−1^ upon iv administration)^6^ 6Determined by INTOX, 375, Urawade, Pirangut Urawade Road, Tal. Mulshi, Dist. Pune, INDIA, in GLP compliance with regulators from India and Europe..

In figure 1, the structure of the RGD motif is also reported. The strong structural similarity between RGD and AGMA1 repeating unit, together with the cationic properties of the latter, might explain the peculiar properties of AGMA1 as regards full biocompatibility, intracellular internalization and trafficking, ability of acting as transfection promoter [11–13] and promoting nerve regeneration in the form of crosslinked hydrogel scaffolds [14–17].

**Figure 2.**
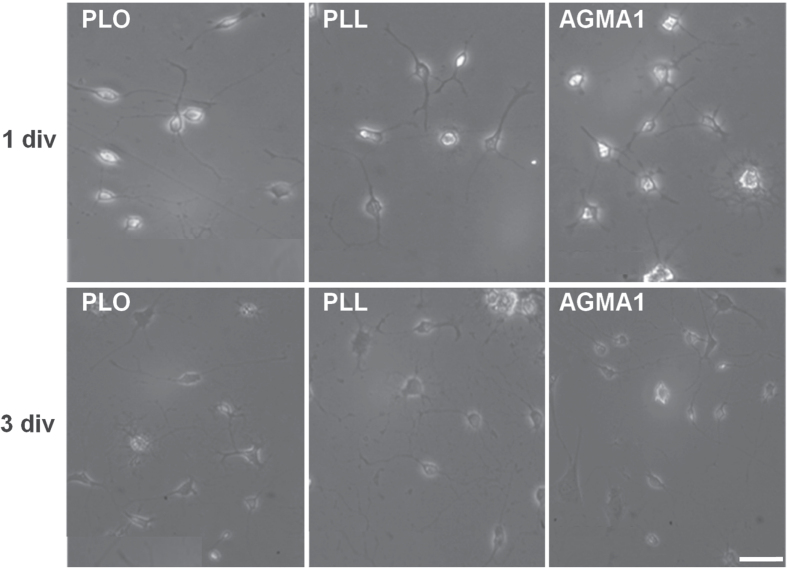
Representative bright field microscopy images of primary rat microglia grown on PLO, PLL and AGMA1. Scale bar = 25 *μ*m.

Based on this premise, the ability of AGMA1-coated surfaces to promote neural cell adhesion was first assessed. Different primary cell types from rat brain were cultured on AGMA1, and the results compared with those of cells cultured under the same conditions on conventional substrates. Primary microglia cultured on AGMA1 were first morphologically compared with those growing on PLO- and PLL–coated substrates (figure 2). Bright field microscopy examination at 1 and 3 days in culture on the three substrates showed the same typical resting ramified morphology, whilst the lack of ameboid-shaped cells excluded the occurrence of activation phenomena.

**Figure 3.**
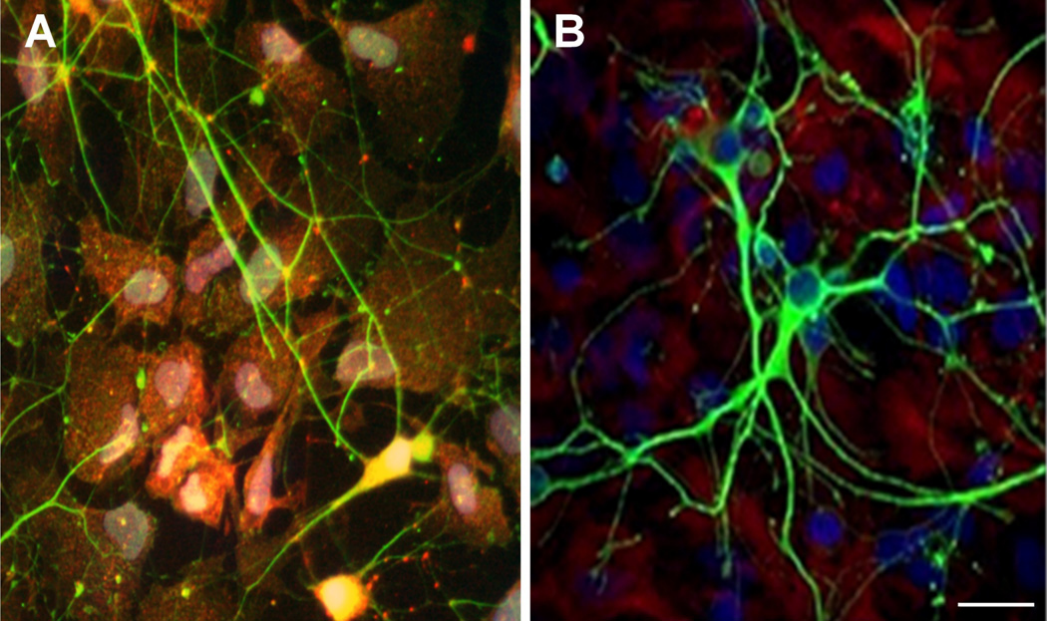
Representative confocal microscopy pictures of primary mixed coculture neurons-astrocytes grown on AGMA1 (A) and PLL (B). Scale bar = 25 *μ*m.

Mixed cultures of primary astrocytes and neuronal cells, stained for the astrocytic marker glial fibrillal acidic protein (GFAP) and for the neuronal marker *β*-III tubulin were morphologically undistinguishable from cells grown on poly-L-lysine, with astrocytes forming a well distributed monolayer and neurons creating networked connections on top of the glia (figure 3).

**Figure 4.**
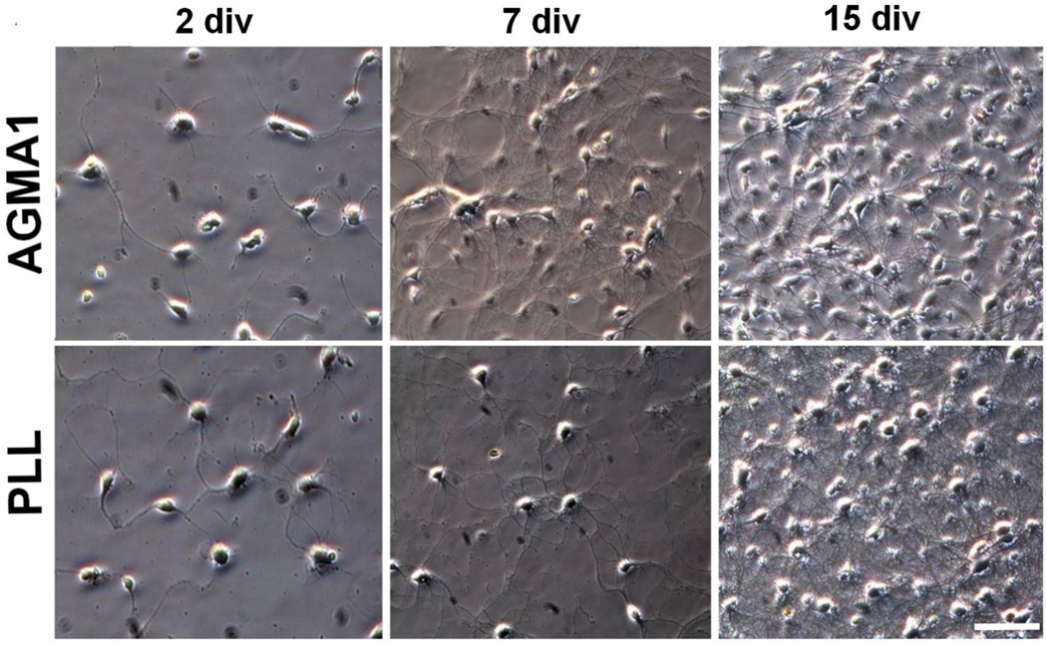
Representative bright field microscopy pictures at different times of primary rat hippocampal neurons grown on AGMA1 and PLL. Scale bar = 25 *μ*m.

A more in-depth analysis was then carried out on primary neuronal cultures. Primary neuronal cell cultures recapitulate the morphological, synaptic and neurochemical features of their *in vivo* counterparts [21]. Therefore, the suitability of a substrate is properly assessed following the development and maturation of the neurons with time. Using PLL as substrate, hippocampal neurons become appropriately polarized, develop extensive axonal and dendritic arbors and form numerous, functional synaptic connections [21–23]. In the present work, primary rat hippocampal neurons were cultured on AGMA1- and PLL-coated coverslips for different time periods and their *in vitro* development followed. On both substrates, hippocampal neurons showed similar steps of maturation with neurite branches extending at the initial stages of neuronal maturation, sometimes with a more pronounced network observable at 7 div in neurons grown on AGMA1 compared with those grown on PLL. Both neuronal cultures reached a mature, well-developed network at 15 div (figure 4).

**Figure 5.**
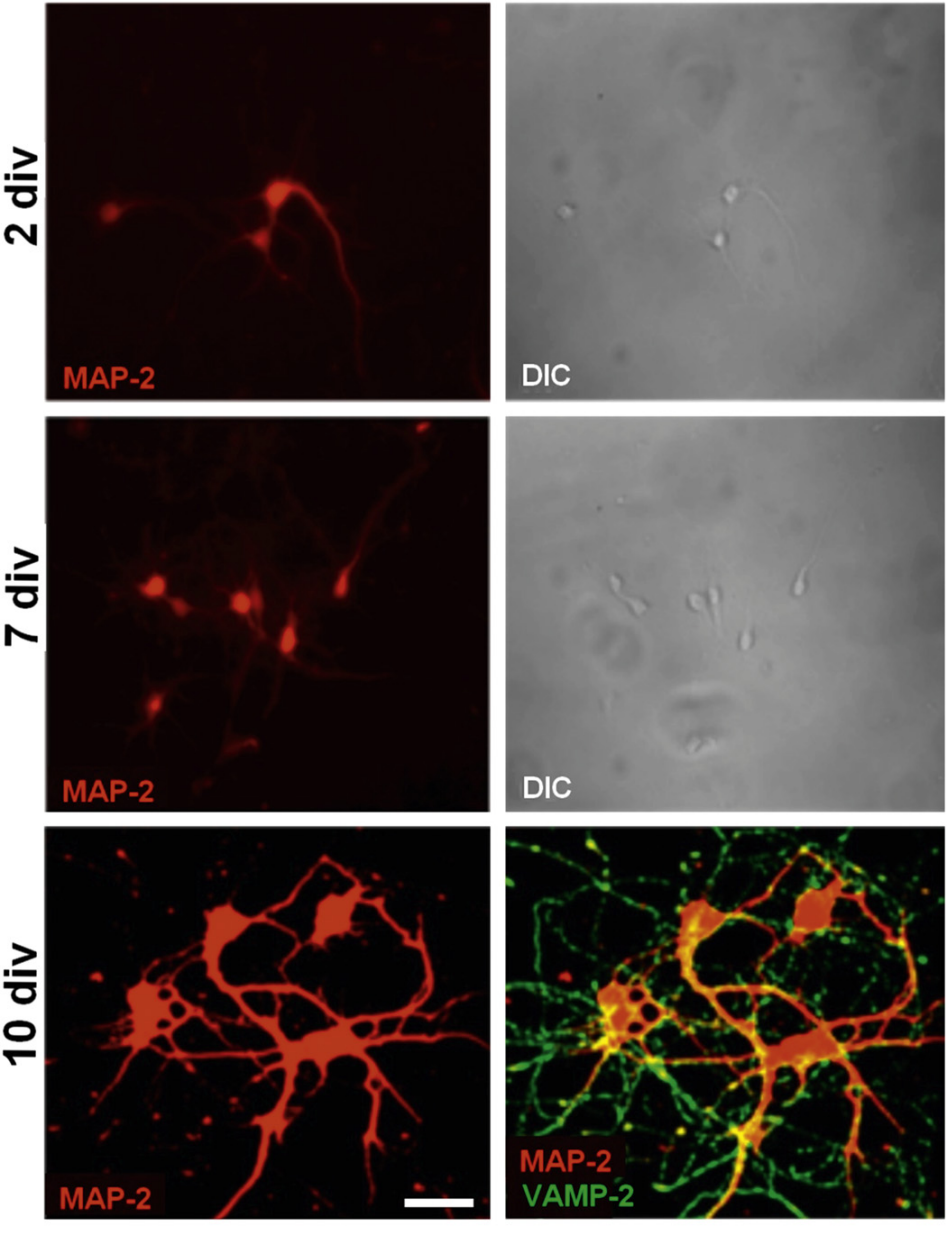
Representative confocal microscopy pictures of primary hippocampal neurons stained with early marker MAP-2 at 2, 7 and 10 div and with both MAP-2 and synaptic vesicle associated marker VAMP-2 at 10 div. Differential interference contrast (DIC) images at 2 and 7 div. Scale bar = 25 *μ*m.

Subsequent morphological observation carried out by confocal microscopy at different stages of development gave evidence of the capability of primary developing neurons to branch, as assayed by selective staining with the somatodendritic marker microtubule associated protein 2 (MAP-2), and establish mature, functional synapses, as assayed by selective staining at 10 div for the vesicle associated membrane protein (VAMP-2) that is involved in synaptic vesicle fusion (figure 5).

**Figure 6.**
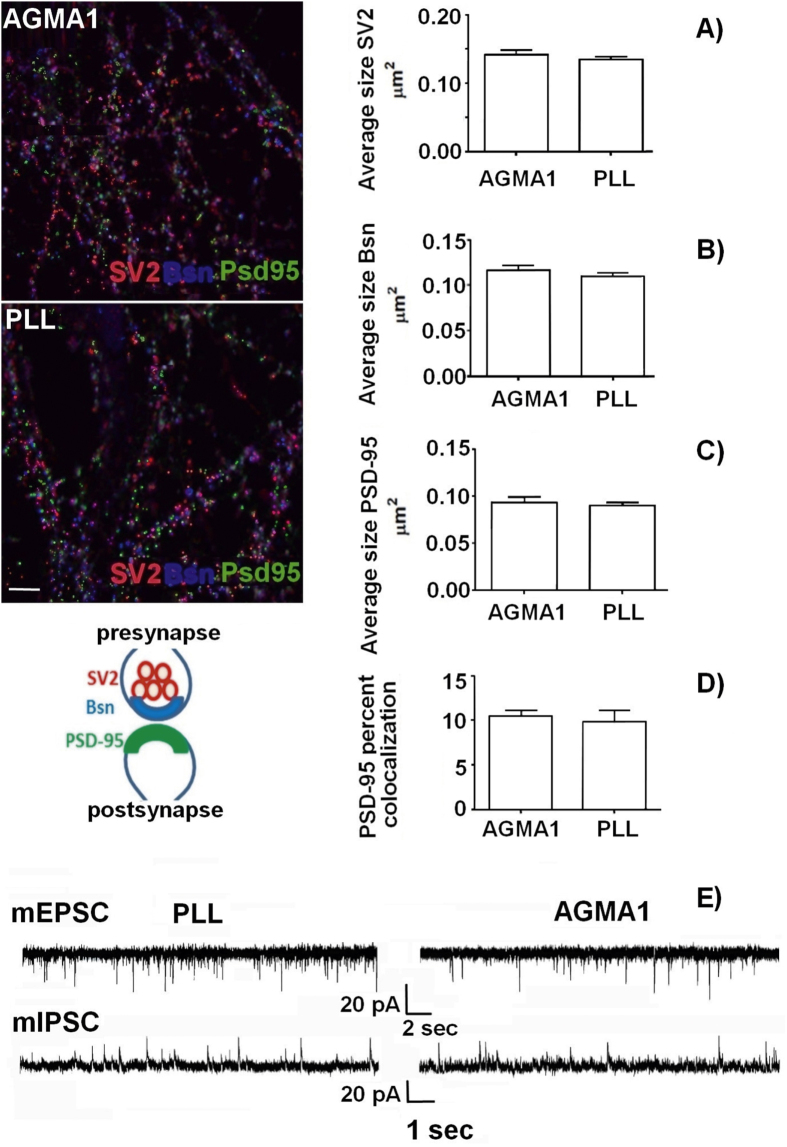
Immunofluorescence staining of 16 div primary hippocampal neurons plated on AGMA1 and PLL with synaptic markers SV2 (red), Bassoon (Bsn, blue) and PSD-95 (green). Quantitative analysis: average size of SV2 (A), Bassoon (B) and PSD-95 (C) puncta. (D) Percentage of area of PSD-95 puncta colocalized with SV2 and Bassoon vs total area of PSD-95 puncta. No significant difference in all parameters is detectable between cultures plated on AGMA1 or PLL. The cartoon illustrates the relative localization of the three markers at the synapse. Scale bar = 25 *μ*m. (E) Representative traces of mEPSCs and mIPSCs recorded from 16 div hippocampal neurons plated on PLL substrate or AGMA1 showing the occurrence of miniature events in both experimental conditions, indicating that neurons cultured on AGMA1 are characterized by spontaneous activity comparable to that of neurons grown on PLL coated medium.

Immunocytochemical staining for the synaptic vesicle protein (SV2A), for the active zone component Bassoon and for the postsynaptic scaffold protein (PSD-95) were performed at 16 div to investigate whether synaptic contacts formed by primary neurons on AGMA1 and poly-L-lysine were correctly differentiated. Neuronal development and synaptogenesis coincided with a correct localization of the three markers, which appeared juxtaposed (figure 6 cartoon). The size, density and percentages of co-localization of these markers were then quantified (figures 6(A)–(D)).

After two weeks, a dense network of synaptic contacts were formed by neurons plated on both substrates with the presynaptic markers SV2 (red) and Bsn (blue) being juxtaposed to the postsynaptic marker PSD-95 (green) (see figure 6). A quantitative analysis of SV2, Bsn and PSD-95 size (figures 6(A)–(C)) revealed that no difference existed between AGMA1 and PLL, while the quantitative co-localization analysis (figure 6(D)) further confirmed that synaptic contacts were correctly formed on both substrates.

To further investigate whether synapses formed by neurons plated on AGMA1 were in fact functional, electrophysiological recordings of miniature excitatory (mEPSCs) or inhibitory (mIPSCs) currents were performed, holding neurons at the reversal potential for *γ*-aminobutyric acid or glutamate-mediated responses (−70 and +5 mV, respectively) in the presence of 1 mM tetrodotoxin. Figure 6(E) shows that neuronal networks formed on AGMA1 and PLL displayed comparable excitatory and inhibitory neurotransmission, demonstrating that the synaptic contacts formed are fully functional.

## 4. Conclusions

The present investigation was aimed at ascertaining the potential of a biocompatible, water soluble and RGD-mimetic PAA named AGMA1 as adhesion, proliferation and differentiation promoter of primary brain cells when surface-adsorbed on cell culturing substrates. All experimental results prompted the conclusion that the AGMA1 performance in this respect is comparable to that of conventional polycations such as PLL. The primary astrocytes and neuronal cell co-cultures run on the two substrates proved undistinguishable on comparative morphological analysis. Moreover, electrophysiological recording of the spontaneous activity of neurons grown on AGMA1 and poly-L-lysine, combined with morphological analysis, demonstrated that in both cases the neurons not only differentiated axon and dendrites, but also established perfectly functional synaptic contacts.
